# Interpreting results from biomarker-guided clinical trials: avoiding “negative” or “failed” terminology

**DOI:** 10.1186/s12916-025-04035-9

**Published:** 2025-05-28

**Authors:** Nurulamin M. Noor, Haiyan Zheng, David S. Robertson, Joshua Savage, Christina Yap, Andrea L. Jorgensen, James M. S. Wason, on behalf of the MRC-NIHR Trial Methodology Research Partnership (TMRP) Precision Medicine Working Group

**Affiliations:** 1 Department of Gastroenterology, Cambridge University Hospitals NHS Foundation Trust, Cambridge, UK; 2 Department of Medicine, School of Clinical Medicine, University of Cambridge, Cambridge, UK; 3 Medical Research Council Clinical Trials Unit, University College London, London, UK; 4 Department of Mathematical Sciences, University of Bath, Bath, UK; 5 MRC Biostatistics Unit, University of Cambridge, Cambridge, UK; 6 Cancer Research UK Clinical Trials Unit (CRCTU), University of Birmingham, Birmingham, UK; 7 Clinical Trials and Statistics Unit, The Institute of Cancer Research, London, UK; 8 Department of Health Data Science, University of Liverpool, Liverpool, UK; 9 Biostatistics Research Group, Population Health Sciences Institute, Newcastle University, Newcastle Upon Tyne, UK

**Keywords:** Biomarkers, Biomarker-driven, Biomarker-guided, Biomarker-stratified, Precision medicine, Clinical trials

## Background

Well-conducted clinical trials provide data to support decision-making for healthcare policy, guidelines, and clinical practice [1]. In recent years, there has been rapid development in genomics and development of novel therapeutics, which have provided hope for a more personalised or precision approach to healthcare. One main method that has been proposed by which precision medicine could be delivered is using validated biomarkers to enable patient stratification.

Following robust assessment of biomarkers for analytical validity and clinical validity, it is widely accepted that the next key step is for clinical utility to be demonstrated [2]. There have been a multitude of biomarker-guided clinical trial designs reported in the literature to assess for clinical utility of a biomarker for patient stratification (as opposed to a surrogate endpoint), including those using a fixed-sample design or adaptive design [3, 4].

With the increased complexity of biomarker-guided trial designs, there is potentially a greater chance for misunderstanding and misinterpretation of outcomes. Therefore, education and training are required to enable appropriate interpretation of such trials [5]. In this article, we summarise key principles to consider when evaluating the results of biomarker-guided clinical trials, including how to appropriately interpret results from such trials, and we stress the importance of appropriate terminology and wording to reflect understanding of these trials.

## Main text

There have been multiple lessons learned from the early implementation of biomarker-guided designs [6]. Although many of the processes of delivering biomarker-guided trials are similar to delivering non-biomarker-guided trials, there are some important additional considerations. Moreover, multiple biomarker-guided trial designs can be appropriately applied to answer questions of clinical utility, therefore there is no single “correct” trial design to use in all circumstances and all disease areas [7].

A critical aspect worth emphasising, and which distinguishes non-biomarker-guided trials from biomarker-guided trials, is that the purpose of most biomarker-guided clinical trials is to assess whether a biomarker demonstrates clinical utility or not—it is not primarily to demonstrate whether a treatment intervention is effective or not [7]. Specifically, demonstrating a biomarker does not have clinical utility (no matter whether the treatment intervention is effective or not) is an important finding as this allows researchers to focus resources elsewhere to answer other appropriate clinical research questions.

Recent examples from the literature include a biomarker-stratified clinical trial called PROFILE (Predicting outcomes using a molecular biomarker for patients with Crohn’s disease) [8], which sought to assess a blood-based prognostic biomarker for patients newly diagnosed with Crohn’s disease, a chronic inflammatory condition of the gastrointestinal tract [8]. The trial used a biomarker-stratified approach with the primary outcome measure being a biomarker-treatment interaction effect, where it was anticipated that early, effective therapy might be more effective only in a subgroup of high-risk patients. Given the need to evaluate an interaction effect, the trial had a much larger estimated sample size than would have been required if only evaluating a treatment effect. Importantly the trial recruited to the target sample size, and despite promising credentials of the biomarker from prospective observational studies, the prognostic biomarker in the interventional trial did not demonstrate clinical utility (lack of statistical significance for a biomarker-treatment interaction effect). An important additional finding (likely explaining the lack of utility of biomarker-based stratification), was a large benefit of early, effective therapy in the majority of patients—with the results supporting a widespread change in clinical practice.

In addition, the K-Umbrella GC (Korean-Umbrella Gastric Cancer) trial [9], sought to assess a range of targeted second-line medication treatments using a biomarker-guided approach within an umbrella trial design. A total of 318 patients were enrolled and received either open-label biomarker-guided treatment or the current standard-of-care treatment. The primary endpoint was progression-free survival. Similar to the above example, again no clinical utility was demonstrated for the biomarker-guided approach compared to the control group when considering survival outcomes; however, the trial showed that a biomarker-guided approach within an umbrella trial design could be successfully delivered and that biomarker-guided treatment was a feasible future strategy for second-line treatment in gastric cancer.

Although both of the above examples would be considered by many in the respective fields as being clinically impactful, it is sobering to reflect that others including peer reviewers may interpret these trials as “negative” or “failed”. Even before the advent of biomarker-guided trials, the terminology of “negative” or “failed” clinical trials has been used to describe clinical trials that do not meet a pre-specified primary outcome of interest—typically lack of efficacy of interventional therapies. These “negative” results have often been framed as scientific failures [10]. Indeed, the historical framing of clinical trials as “negative” may be one of the contributors to the problem of publication bias, whereby results from trials can go unreported and unpublished [11].

However, we believe the dogma of describing trials as “negative” or “failed” is outdated and unhelpful and we would highlight the sizeable impact and contribution from clinical trials demonstrating interventions which were not found to be “positive”. Indeed, the problem of publication bias highlighted above may be even more pronounced in the context of biomarker-guided trials. Failure to publish results from clinical trials of biomarkers not demonstrating clinical utility would pass up the opportunity to provide important information to the academic community, potentially allowing further unnecessary research to continue unchecked.

We propose that researchers, reviewers and editors of journals should push back on the inappropriate use of “negative” or “failed” terminology used to describe biomarker-guided clinical trials. Each clinical trial assessing a biomarker for clinical utility should be judged on its own merits in terms of robustness of design, conduct, analysis and reporting.

Importantly, for biomarker-guided trials, there should be sufficient evidence that a biomarker would plausibly provide utility based on evidence to date. A critical subsequent step is ensuring that the sample size of a biomarker-guided trial would be sufficiently powered to demonstrate a biomarker is useful. Thorough and appropriate design, conduct and analyses of the biomarker-guided trial itself should then seek to minimise biases. To support the statistical rigour required for biomarker-guided trials we would recommend the following principles could be applied: development of the statistical analysis plan alongside the protocol, use of the estimand framework to ensure clarity on the exact question(s) seeking to be answered from the trial, and ideally having appropriately qualified and experienced statisticians and independent oversight committee members with experience on previous trials with biomarker incorporation.

If all of the above factors are accounted for, then even for clinical trials not demonstrating the clinical utility of a biomarker, we assert that such a trial should be considered a “success”—given that the research question has been answered. In this regard, “lack of success” would only be applicable to describe a biomarker-guided trial that fails to provide answers to any questions—this may occur for reasons such as minimal recruitment or early withdrawal of funding. We strongly suggest that as a field we should move away from “positive/negative” or “successful/failed” terminology, and instead would advocate for terminology to refer to whether the research question seeking to be answered in a biomarker-guided trial has been answered or not with the corresponding results provided.

## Conclusions

There are a rising number of validated biomarkers in medicine. Even for biomarkers with promising credentials in an observational study, the benefits of biomarker-guided trials to assess clinical utility are well illustrated by the above examples. A crucial aspect for progress will be to increase understanding both around the conduct and interpretation of findings from such trials. This should help inform which biomarkers should or should not be incorporated into routine clinical care.

## Data Availability

No datasets were generated or analysed during the current study.

## Abbreviations

K-Umbrella GC: Korean-Umbrella Gastric Cancer
PROFILE: Predicting outcomes using a molecular biomarker for patients with Crohn’s disease
